# Modelling Socio-Environmental Sensitivities: How Public Responses to Low Carbon Energy Technologies Could Shape the UK Energy System

**DOI:** 10.1155/2014/605196

**Published:** 2014-01-27

**Authors:** Brighid Moran Jay, David Howard, Nick Hughes, Jeanette Whitaker, Gabrial Anandarajah

**Affiliations:** ^1^Institute for Energy Systems, School of Engineering, University of Edinburgh, King's Buildings, Edinburgh EH9 3JL, UK; ^2^Centre for Ecology and Hydrology, Library Avenue, Lancaster, Bailrigg LA1 4AP, UK; ^3^Centre for Environmental Policy, Imperial College, 15 Prince's Gardens, South Kensington, London SW7 2AZ, UK; ^4^UCL Energy Institute, University College London, Central House, 14 Upper Woburn Place, London WC1H 0NN, UK

## Abstract

Low carbon energy technologies are not deployed in a social vacuum; there are a variety of complex ways in which people understand and engage with these technologies and the changing energy system overall. However, the role of the public's socio-environmental sensitivities to low carbon energy technologies and their responses to energy deployments does not receive much serious attention in planning decarbonisation pathways to 2050. Resistance to certain resources and technologies based on particular socio-environmental sensitivities would alter the portfolio of options available which could shape how the energy system achieves decarbonisation (the decarbonisation pathway) as well as affecting the cost and achievability of decarbonisation. Thus, this paper presents a series of three modelled scenarios which illustrate the way that a variety of socio-environmental sensitivities could impact the development of the energy system and the decarbonisation pathway. The scenarios represent risk aversion (DREAD) which avoids deployment of potentially unsafe large-scale technology, local protectionism (NIMBY) that constrains systems to their existing spatial footprint, and environmental awareness (ECO) where protection of natural resources is paramount. Very different solutions for all three sets of constraints are identified; some seem slightly implausible (DREAD) and all show increased cost (especially in ECO).

## 1. Introduction

Over the past decade, the UK government has become increasingly aware that climate change and energy security are urgent issues that will drive energy system change [1, 2]. Reducing the UK's climate change impacts through decarbonisation while also achieving a secure, resilient energy system depend on both strategies to reduce energy demand and the deployment of low carbon energy technologies. However, both the academic and policy communities have focused their attention on developing and deploying technologies to achieve decarbonisation. There has typically been less attention given to the consequences of successfully voiced public objections to low carbon technologies and more generally how this will influence the development of the whole energy system. Yet, this is an important factor in shaping the possible pathways to decarbonisation because public resistance to certain technologies will alter the portfolio of options available. The UK Energy Research Centre (UKERC) modelled future scenarios of the UK energy system in the Energy 2050 project [3]. It explored how public acceptance, as motivated by particular socio-environmental sensitivities, could affect the deployment of certain resources and technologies with consequent impacts on the whole energy system.

Some scenarios in Energy 2050 looked at the potential for accelerated development of low carbon energy supply technologies to alter the pathways to UK energy system decarbonisation [4, 5]. However, those accelerated technology development (ATD) scenarios do not reflect the potential for public responses to constrain the deployment of energy supply technologies. These socio-environmental constraints could shape the energy system as well as the possible pathways to decarbonisation in UK.

Only recently has research started to be undertaken about how public attitudes and responses could affect the energy system at a system-wide level (e.g., [6]). There are studies that look at individual technologies, for example, nuclear power [7], carbon capture and storage [8], and biomass [9], but very few that look across technologies as Carr-Cornish et al. [10] did for Australia. Poortinga et al. [11] investigated lifestyle and motivational factors that influence both demand and supply of energy but did not fully explore the impact on the whole energy system.

This paper addresses the gap in knowledge by examining how people's responses to particular energy technologies (as determined by certain socio-environmental sensitivities) could play a role in shaping the UK energy system. Socio-environmental sensitivities in this paper are illustrated in three different simplified scenarios which represent how a given public concern/motivation could manifest itself in the form of resistance to certain technologies and resources.

The scenarios, named NIMBY, ECO, and DREAD (described in the next section), are illustrations of the types of public responses that could be seen given particular simplified motivations. The motivations behind each of the three variant scenarios are qualitatively characterised based on existing literature about public attitudes towards technologies and case studies of particular developments and siting conflicts. They do not, however, reflect the complexities of public opinions in the real world. In reality, these responses would not be seen in isolation and unchanging over time, as is the case in these scenarios.

Likewise, public opinion is not always a barrier; it can also be an important enabling factor. However, the scenarios provide a useful opportunity to examine how different types of public responses could affect the possible pathways to decarbonisation of the energy system. Without considering the possible social acceptance limitations, UK could face unexpected disruptions on the pathway to decarbonisation because socio-environmental sensitivities could be important determinants of whether, how, and at what cost UK achieves decarbonisation. As such, socio-environmental sensitivities require further research to better understand the potential implications on the energy system. The debate in Britain about the exploitation of shale gas and the impact of demonstrations is an example of public sensitivity to novel technology that will at least add delays and expense to exploitation but may prevent its development.

## 2. Methodology

The UK energy system was modelled in UKERC Energy 2050 [3] using MARKAL (UK MARKAL model), a market allocation economic model that uses linear programming to reflect change over 5-year time steps. It is described as “bottom-up” containing detailed information about different energy platforms and sources and portrays the whole energy system including the energy service demands of the whole economy. The elastic demand version of the model (UK MARKAL MED), where demands respond to supply price changes under policy scenarios, has been used in UKERC Energy 2050 studies. In this version of the model, higher energy prices in general drive greater demand reductions. However, the cost savings achieved from reducing demand and consuming less fuel (gas, electricity, petrol, etc.) are balanced against the cost of demand reduction as defined in MARKAL-MED, as a result of foregoing the energy services in question (they enjoy slightly less heating, electrical services, or transport). The model optimises this balance based on the value of the energy services, compared to the cost of supplying them. In other words, it optimises for the sum of producer plus consumer surplus. Details of the UK version of the model and its uses can be found in papers by Strachan and his colleagues [12, 13].

Using one of the core UKERC Energy 2050 scenarios, the low carbon (LC) scenario, as a baseline scenario, three variant scenarios were developed which modelled distinct storylines about how people could respond to and constrain the deployment of key low carbon energy supply technologies and resources. The LC baseline scenario is based on firm and funded policies as of the Energy White Paper 2007 and achieves 80% decarbonisation by 2050 [14]. Accordingly, all the variant scenarios described in this paper also achieve 80% decarbonisation by 2050. The variant scenarios for the socio-environmental work are named NIMBY, ECO, and DREAD. The qualitative definitions and storyline of the scenarios are based on the interpretation of the literature on public attitudes and responses to energy technologies and case studies of particular developments and siting conflicts. But the scenarios are necessarily simplified. The scenarios largely focus on the electricity sector technologies and resources, with some exceptions where there are important crossovers between sectors (such as bioenergy).

Using published literature as well as consultation with experts, each scenario storyline was then translated into quantitative impacts (constraints) on the deployment of particular technologies. The impacts on the technologies described in this work are not meant to be comprehensive or “scientifically objective” statements; instead, they reflect a hypothesised vision of how the general public under certain conditions might respond to the technology.

Using these new technology and resource constraints, each scenario was then modelled in the MARKAL-MED energy system model. In this project, the MARKAL model is used as a tool to explore the issue of socio-environmental constraints and the modelling results are meant to *illustrate* relevant issues for further exploration; it is not meant to be a prediction of the future. MARKAL represents a techno-economic view of the energy system which includes a high level of detail on technology and resource costs and availability. However, the model does not include any detail on the social conditions in which the energy system is set, such as, in this case, public attitudes and the constraints that could be imposed by public objections. By representing these socio-environmental constraints in the model, this work sought to use the MARKAL model in a novel way.

The resulting changes to the energy system are evaluated in terms of the overall change in energy system make-up (such as technology selection and demand reduction) and the costs of the energy system and decarbonisation. The potential impacts of accelerated development of low carbon energy supply technologies on these socio-environmental scenarios are then considered in a discursive manner.

## 3. Socio-Environmental Scenarios

### 3.1. NIMBY Scenario Definition

The NIMBY scenario represents a storyline in which the public objects to certain energy developments when they perceive the development to have direct negative impacts on their lifestyle and community. The primary direct impact considered in this scenario is visual intrusion which many studies have cited as a key reason for people's opposition [15–17]. Accordingly, in the NIMBY scenario, people exhibit a strong resistance to major changes in their landscape. Devine-Wright [18] has described this type of opposition “as a form of place-protective action, which arises when new developments disrupt pre-existing emotional attachments and threaten place-related identity processes.” Gee [19] highlights that people's perceptions of their landscape play a role in shaping attitudes: the type of perception of the landscape that would play a key role in this scenario is that which recognises the recreational, spiritual, and aesthetic benefits that people get from their environment. Thus, in NIMBY, people's attachment to their landscape is the primary driver of opposition to the place-disruption that they believe would occur with a given energy development [20].

Based on this definition of NIMBY as a form of place-protective action with strong concern for the visual landscape, the NIMBY scenario has strong constraints imposed on the deployment of technologies that are visually obtrusive. This included both technologies that have in the past been objected to and new technologies where large visual impacts are anticipated. Accordingly, where the technology and infrastructure already exist, further developments will generally be accepted. However, certain technologies will be limited in places where that type of development is unfamiliar and raises objections based on the visual landscape impact.

In the NIMBY scenario, the definition of people's landscape is not exclusive to a person's immediate local area in terms of distance. Studies have shown that proximity to a development is not always directly correlated with support or opposition [21, 22]; rather, in this scenario, a person's landscape is more broadly defined to include places to which that person has some type of attachment. People may have attachments to their local home area as well as places such as holiday spots, national parks, or other landscapes of special interest. In addition, studies suggest that a person's landscape or “backyard” may not even stop at the coastline, that people can sometimes consider offshore locations as part of their landscape [23].

It should be noted that this project's definition of NIMBY is very different from the classic and highly contested description of NIMBY, defined as “not in my backyard.” This classic definition envisions the NIMBY objection as opposition to a project which is not based on opposition to the technology itself but rather opposition to a particular project based on self-interest. Some describe true NIMBYs as expressing positive attitudes towards a technology while also expressing free rider preferences [15]. Many studies have suggested that this classic NIMBY definition is an inaccurate and erroneous term because it fails to reflect people's true motivations and masks a wide range of different motivations [16, 24–26]. Further, other studies have shown little or no evidence for the classic self-interest definition of NIMBYism [15, 27–29]. It appears that NIMBY has become a pejorative term often used to dismiss people's valid concerns. Although the term NIMBY is contested in academic literature, NIMBY is still used as the title for this scenario in large part because it has become a recognisable term in popular usage. However, to avoid the pitfalls of the term NIMBY, it has been carefully defined above as a form of place-protective action based on negative visual landscape impacts.

### 3.2. NIMBY Scenario Quantification of Impacts

In the NIMBY scenario, the deployment of onshore wind power is limited based on the visual impact of the turbines, which has been identified as a key reason behind people's objections to particular wind farms [15, 16, 21, 30]. The NIMBY scenario assumes such a strong opposition to wind developments based on visual impact that no new onshore wind applications receive planning approval. However, a number of the projects which are already under construction or have received planning consent are allowed to be built (figures for this based on BWEA, [31]). This constraint translates to a total of 8.9 GW of onshore wind power capacity allowed in the MARKAL model.

Offshore wind developments in the NIMBY scenario are also subject to public objections based on visual impact. Many studies have found that visual impact is a key factor influencing people's opinion of wind farms. Hagget [32] found that simply siting wind turbines offshore does not automatically solve problems of visual impact. Therefore, in NIMBY, developments are only allowed beyond a 12 nautical mile buffer where the turbines would be less visible and there could be less public objections [23, 33–35]. Using data from the Department of Energy and Climate Change (DECC)'s Strategic Environmental Assessment Report [34], the available offshore wind power beyond a 12 nautical mile buffer zone is taken to be 80 GW. This was used as the total constraint on offshore wind power for the NIMBY scenario in MARKAL.

The Severn tidal barrage is not allowed in the NIMBY scenario because in this storyline, it is perceived as a negative direct impact on the community. There are concerns over the landscape impact of the barrage such as the potential shifts in landscape type (such as mudflats and marshes), changes to local sense of place-identity and place-attachment, and changes to historic ports.

Nuclear power plants are not allowed to be sited in new locations in the NIMBY scenario. However, in areas around existing nuclear power plants, the nuclear plant is assumed to already be a feature of the landscape and community, and therefore, in this scenario, the local community would be likely to be willing to accept further nuclear power. Studies have shown that people living near nuclear facilities express higher support for increasing nuclear power in the future [36], albeit perhaps transitory acceptance, with an ebb and flow of concerns [37]. This local acceptance combined with a general “reluctant acceptance” of nuclear power to help combat climate change [38] means that in the NIMBY scenario, existing nuclear power plants are allowed to be rebuilt at the end of their lifetime and in some cases expanded (at sites that already host multiple reactors). Using an assumption that new 1600 MW EPR reactors would be built on existing commercial nuclear power sites, this scenario allows for up to 30.4 GW of nuclear power.

In the NIMBY scenario, coal carbon capture and storage (CCS) is constrained by public objections to the direct landscape impact of new power plants, capture plants, and corresponding infrastructure such as pipelines and storage facilities. As such, coal CCS is only allowed at a few existing coastal power plant sites or other major existing industrial sites. CCS technology appears to have low levels of public understanding [39] and is unfamiliar to most people. Only a few studies have examined attitudes to CCS and they have found some negative attitudes towards storage and pipelines for CCS [40, 41]. Another study suggests that there may be differing opinions depending on the type of storage being proposed (i.e., geological or oceanic) as well as the context in which the technology is considered [42]. This is taken to suggest that in a NIMBY scenario, CCS would be constrained. Yet, there would likely be a few coastal sites not subject to NIMBY objections. So, for the NIMBY scenario, roughly seven sites were considered to be consentable. With this estimation, up to 10.5 GW of coal CCS installed capacity would be allowed.

Bioenergy would be limited in the NIMBY scenario due to people's concerns over large areas of land being switched to growing crops to be used for bioenergy. Bioenergy here means biomass for heat and power production as well as in the form of biofuels for transport. Switching large amounts of land over for the purpose of growing biomass for bioenergy would noticeably alter the landscape character and thus would be subject to NIMBY concerns. Heiskanen et al. [43] suggest that divergent interests in land use are one of the major conflicts that can be found in case studies of public acceptance of bioenergy. Therefore, an analysis was done using a spatial mapping approach (with Joint Character Areas) to determine what percentage of the UK productivity might be available/allowable for bioenergy production under a NIMBY scenario. This analysis suggested that under strong NIMBY concerns, only 37% of the UK productivity would be allowed to be cultivated by traditional crops for energy use.

In addition, second generation, dedicated energy crops such as Miscanthus and willow (which are unfamiliar and look quite different in the landscape from traditional crops) were deemed to be unacceptable under NIMBY conditions and thus none were allowed in the NIMBY scenario. So, only first generation, traditional crops are allowed for energy production.

### 3.3. ECO Scenario Definition

The ECO scenario represents public objections to certain technologies and resources based on the public's perception of negative impacts on the natural environment and ecosystem services. In the ECO scenario, low carbon technologies are not simply justified on the grounds of the reduction in carbon emissions; other environmental impacts are considered to be important as well. Some studies of energy projects, such as Firestone and Kempton [44] who looked at the Cape Wind offshore wind project in USA, have suggested that the public's objections are largely based on a perception of negative environmental impact. This reflects a “green on green” clash of environmental values between the protection of the environment through increased use of low carbon energy technologies and the other environmental damage that could be caused by deployment of those low carbon technologies [21]. This contradiction between different environmental values makes the deployment of certain technologies problematic [15].

The specific impacts included in this scenario may deviate from an expert's opinion in some cases but this is appropriate here because this scenario aims to represent resistance to key ecological impacts *as perceived by the general public*. These public perceptions are informed by media coverage, high profile scientists and NGOs, friends, and family as well as the general level of knowledge about the impact. The public can be seen to have differing levels of trust in key actors of an energy development based on the perceived competence and motivation of those actors [40]. These varying levels of trust, understanding, and media coverage would all be expected to shape the public opinion of the environmental impact of the project.

### 3.4. ECO Scenario Quantification of Impacts

In the ECO scenario, the public objects to some proposed onshore wind farms due to concerns about the impact of the project on bird and bat mortality and damage to the land around the turbines (for instance peat bogs and construction damage). Studies have shown that concerns about birds can have a direct impact on the decision to object to a certain wind project even if it is not expressed in that person's general attitude towards wind power [15]. Yet, not all wind farms are perceived as an ecological threat. Thus, in the ECO scenario there is not a total constraint on the development of onshore wind power; rather, a percentage of developments are assumed to become highly contested on environmental grounds and consequently do not receive the necessary planning approval. In the ECO scenario, this constraint was translated into a total capacity limit of up to 15 GW of onshore wind that would be allowed in UK (based on 25% of the total 20 GW resource available in the MARKAL model being rejected by ECO concerns).

Offshore wind power in the ECO scenario is also assumed to be moderately constrained by public concerns about the potential ecological impact. Yet, these concerns are likely to be lower for offshore wind farms that are located far offshore as early evidence suggests reduced ecological impacts far offshore [34, 35]. Thus, in the ECO scenario, offshore wind development is only allowed beyond a coastal buffer of 12 nautical miles which would limit the potential ecological impacts on, for instance, bird life [34, 35]. DECC [34] has suggested that there is 80 GW of offshore wind power available beyond 12 nautical miles which was therefore used as the constraint on the installed capacity of offshore wind in MARKAL.

The public perception of environmental impacts of wave and tidal power is not very well understood. The general public does not know very much about marine energy devices. In addition, the devices are still being demonstrated and thus there has been little conclusive research on their environmental impact because demonstration projects are not always obligated to carry out an environmental impact assessment [45]. However, there is starting to be more attention paid to the potential ecological impacts of wave and tidal devices. For the purposes of the ECO scenario, it is assumed that as more research is conducted, some negative ecological impacts will be identified—for instance damage to marine ecology due to leaking fluids or other operational features [46]. Accordingly, in the ECO scenario, the general public objects to certain wave and tidal devices as well as objecting to developments in particular sensitive areas. For this exercise, this is estimated as 25% of the wave and tidal resource being unavailable for development due to ecological concerns.

The Severn tidal barrage would not be allowed in the ECO scenario because of public concern that the barrage would damage the environment. A coalition of well-known organisations such as the National Trust, RSPB and WWF opposes the barrage as an environmental mistake; the public in this scenario are assumed to follow these well-respected and high profile organisations and oppose the barrage. Reports from organisations such as the Sustainable Development Commission [47] which found that there could be a serious impact on the environment if the Severn barrage (Cardiff-Weston Scheme) went ahead would also influence public opposition to the barrage. Thus, the Severn barrage is not allowed in ECO.

In the ECO scenario there are strong public concerns about the sustainability merits of imported biomass and biofuels. This has become a popular topic in the media and the general public has heard reports of rainforests being cleared, the high carbon intensity of some crops and scientific reports which call into question the sustainability of biofuels. This coverage influences public perceptions of bioenergy. In this scenario, the public views sustainability issues as manageable for domestic crops but they do not feel confident in the sustainability of imported crops. In order to manage the sustainability of crops domestically, only certain areas of UK would be available for bioenergy production. A spatial mapping analysis using Joint Character Areas suggested that only 11% of the UK productivity could be utilised [48]. Imported biomass and biofuels are completely prohibited in the ECO scenario because of the public concerns. The MARKAL model includes a high level of detail including the distinction between domestic and imported biomass and thus this constraint does limit the resource available in the model. In the ECO scenario, the public also perceive transport biofuels to have the potential to do more damage to the environment than good and thus no transport biofuels (domestic or imported) are allowed in UK.

The final constraint imposed in the ECO scenario is in regard to fossil fuel availability. In ECO, global fossil fuel prices are taken to be much higher than they are in the low-carbon (LC) core scenario because under ECO concerns certain environmentally sensitive areas would not be allowed to be exploited and particularly environmentally damaging methods of extraction would also not be allowed, for example, fracking for shale gas. Therefore, in the ECO scenario, the global price of fossil fuels (coal, gas, and oil) is increased substantially. Domestically, open cast coal mining in UK is deemed too damaging and is not allowed in this scenario.

### 3.5. DREAD Scenario Definition

The DREAD scenario is based on the concept of a “dread” response in which people believe that there are some uncertain, involuntary, and potentially catastrophic risks associated with a given technology [49–51]. Thus, in the DREAD scenario the public perceive certain technologies to pose a serious risk to human health which causes them to categorically reject those technologies.

The determination of the potential risk to human health in this scenario is based on people's perception of the risks rather than statistics of death or other quantitative measures of risk. The perception of risk is based on factors such as voluntariness, dread, knowledge, controllability, and benefits [51]. For instance, despite the fact that more people die in car accidents than in nuclear accidents, there is a greater fear of nuclear accidents. This is largely because the perception of risk of a nuclear accident is very different than a car accident; people feel more control over the risks of driving a car, the risk of a car accident is less potentially catastrophic, and people perceive a more obvious benefit from driving a car which outweighs the risks. With nuclear power, however, the benefits may be less obvious and thus the public are less willing to accept a high level of perceived risk for low levels of perceived benefits.

Thus, in this scenario, deployments of technologies which might invoke a DREAD response from the general public are completely disallowed because strong public opposition to those technologies would prohibit approval of any new projects. This scenario is not making a judgement on whether the public's fears are justified or not; it is simply attempting to represent a highly risk-averse scenario.

### 3.6. DREAD Scenario Quantification of Impacts

In the DREAD scenario, people completely reject new nuclear power developments due to fear of potential catastrophic consequences. Historic nuclear accidents such as Windscale, Three Mile Island, Chernobyl, and Fukushima are, in this scenario, assumed to still have a profound effect on society and on people's perception of the nuclear risk. Despite claims that new nuclear power plants will be safer, in the DREAD scenario, people still exhibit widespread concern over the potential for nuclear accidents and storage safety. According to the psychometric framework, nuclear reactor accidents, radioactive waste, and other associated radioactive risks are classified as highly dreaded [51]. Hinman et al. [52] also report high levels of public concern and dread about the potential for catastrophic accidents and storage of waste products. More recently, Corner et al. [7] in a survey showed that only a minority of people expressed unconditional acceptance of nuclear power and the population remained divided over its acceptability. The Fukushima accident sharply increased public concern about safety (Poumaderé et al. [53]) and caused a number of nations to put nuclear development on hold. Therefore, in DREAD, the public oppose all new nuclear developments so no new nuclear power is built. Existing nuclear power plants continue to operate until the end of their lifetime but do not get lifetime extensions.

The public acceptance of coal CCS technologies is very uncertain but may prove to be a serious barrier to deployment. Some studies suggest that people may object to CCS technology based on the fear of the consequences of the technology on human health, which is a dread response [54]. Singleton et al. [50] use the psychometric theory of pubic risk perception to evaluate the public perception of risk from the geologic storage of carbon dioxide for CCS. Their results show that, although CCS with geologic storage is a new technology, it is seen as a fairly high risk and dreaded; Singletons et al.'s rankings place geologic storage of carbon as higher dread than traditional fossil fuels, but lower than nuclear accidents [50]. Thus, in the DREAD scenario, the public is assumed to have a strong dread reaction to CCS which means that no CCS power plants are allowed in UK.

In the DREAD scenario, the public is assumed to have a dread response to hydrogen and fuel cell technology which completely prohibits any deployments. There is a knowledge gap with regard to the public's understanding and perception of hydrogen and fuel cell technologies [55]. However, there are some persistent public ideas about the dangers of hydrogen; for instance, the vision of the Hindenburg disaster persists despite the evidence that hydrogen was not the critical factor in that disaster. This is an example of how powerfully media exposure can affect public attitudes and how important historical precedent can be to public perceptions. Rhetoric about the dangers of hydrogen such as is seen in Shinnar [56] which suggests that a hydrogen fuel cell car could be made into a bomb by terrorists speaks explicitly to the dread factor. This type of publicity and rhetoric about hydrogen would increase the public's dread response to the technology and likely negatively influence public acceptance. Thus, in the highly risk-averse DREAD scenario, the risks of hydrogen fuel cell technologies are perceived as highly unknown and potentially catastrophic (i.e., highly dreaded). Therefore, no hydrogen and fuel cell deployments are allowed in the DREAD scenario.

## 4. Modelling Results

### 4.1. Different Strategies Employed

When modelled with the additional socio-environmental constraints on technologies and resources, as described in the previous section, each of the scenarios employs different strategies in order to continue meeting the energy system requirements (including achieving 80% decarbonisation by 2050) at the lowest possible cost. There are three key elements of these strategies that can be compared: the distribution of sectoral emissions, technology selection and shifting, and demand reduction.

The first key element of the scenario's differing strategies can be seen in the distribution of sectoral CO_2_ emissions. The three scenarios each take unique approaches to decarbonising the energy system in terms of the order and rate of decarbonisation of the sectors, which could be called the decarbonisation pathway. The scenarios employ different strategies with regard to which sectors of the energy system carry the highest burdens of decarbonisation (transport, industry, etc.). All scenarios initially begin with decarbonisation in the electricity sector but do so to varying degrees (and with different mixtures of technologies and demand reduction as will be discussed shortly); it is worth noting that all three variants decarbonise electricity more aggressively than the core scenario on which they are based due to the constraints set by different sectors.

**Figure 1:**
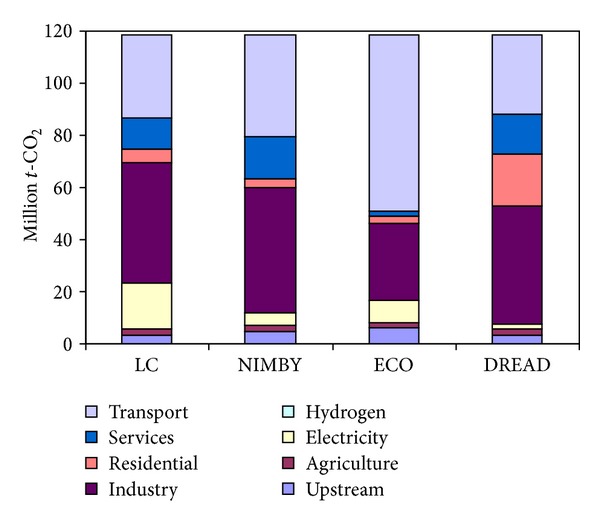
Sectoral CO_2_ emissions in 2050. Scenarios are the low carbon (LC) baseline and the three socio-environmental forms (NIMBY, ECO, and DREAD).

There are significant differences in the scenarios' sectoral breakdown of CO_2_ emissions by 2050 with the ECO and DREAD scenarios showing the greatest divergence from the core LC scenario as seen in Figure 1. In ECO, for instance, the additional constraints such as increased costs of fossil fuels and the prohibition of transport biofuels make the transport sector more difficult and costly to decarbonise. As a result, the system chooses to make greater emissions reductions in other sectors (such as the service and industry sectors) in which there are lower cost decarbonisation options available; the transport sector continues to use diesel and petrol which creates high levels of transport emissions (Figure 1).

In DREAD, the electricity sector is almost completely decarbonised through high levels of deployed wind power to meet electricity demand. The heavy reliance on wind power is due to strong constraints on the availability of other technologies. As a result, the residential sector is able to implement less of the high cost decarbonisation measures and therefore accounts for a larger percentage of emissions than it does in any other scenario (see Figure 1). The residential sector is mostly decarbonised by shifting to low carbon electricity in LC. Since nuclear and CCS are not allowed in DREAD, electricity is no longer a cost effective option to decarbonise the residential sector and model selects other options: decarbonising transport sector.

The second key element of the strategies employed to achieve decarbonisation under the additional socio-environmental constraints is the selection and deployment of different technology mixes. The socio-environmental constraints explored in this work cause the scenarios to have

- lower technology diversity,
- higher demand for electricity generation,
- higher levels of installed capacity and/or
- greater shifts and more exaggeration in the deployment of key energy technologies.

**Figure 2:**
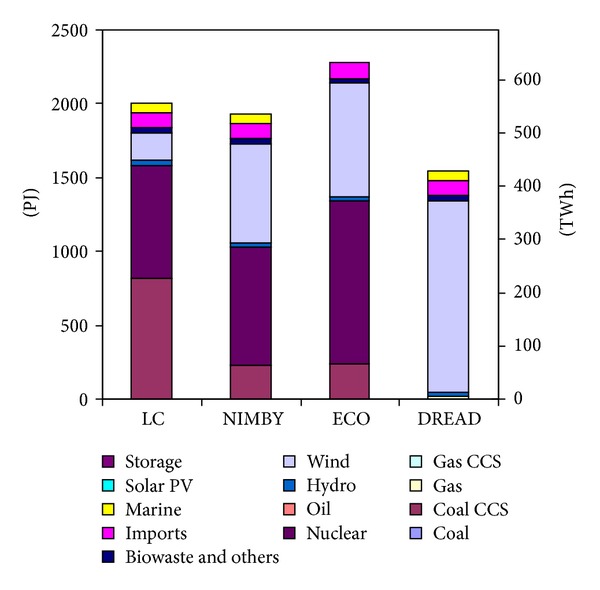
Electricity generation in 2050. Scenarios are the low carbon (LC) baseline and the three socio-environmental forms (NIMBY, ECO, and DREAD).

**Figure 3:**
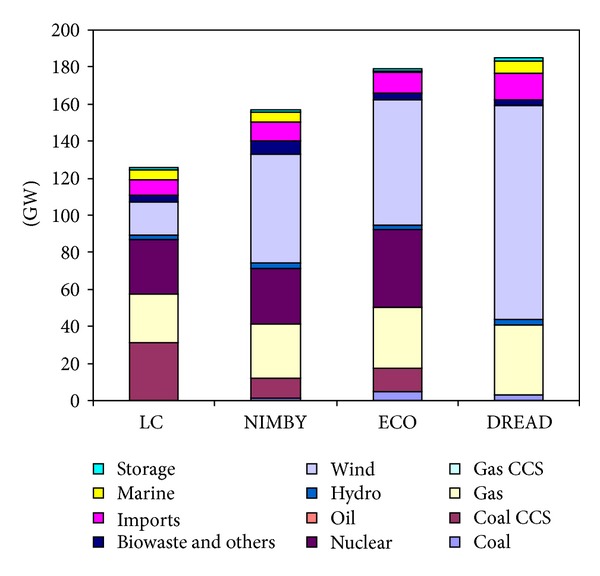
Electricity installed capacity in 2050. Scenarios are the low carbon (LC) baseline and the three socio-environmental forms (NIMBY, ECO, and DREAD).

The differences in supply technology mixes can be seen through the electricity generation and installed capacity of the scenarios (Figures 2 and 3). The key technology shifts highlighted in this paper are dramatic shifts to wind power in DREAD, increased use of nuclear and wind in NIMBY and ECO, and the shifting of limited biomass resources in NIMBY.

Perhaps the most dramatic example of these technology shifts can be seen in the DREAD scenario which has very little diversity in the power sector. In the case of the DREAD scenario the decreased diversity is due to the prohibition of coal CCS or further nuclear power; as a result the model chooses to deploy high levels of wind power to meet the decarbonisation target. By 2050, the DREAD scenario has an electricity sector that is predominately based on wind power. In order to be able to utilise this high wind strategy, the electricity system requires a very high installed capacity with large amounts of installed wind and gas (gas is installed to serve as a backup given wind power's intermittency—although the gas is not highly utilised). As a consequence of this technology shift to high levels of wind power, the scenario requires high levels of demand reduction. Demand reduction is an economically favourable strategy in this scenario because electricity becomes very expensive.

Another potential consequence of DREAD's reliance upon a single electricity generation source could be decreased resilience. Some increased security may come from the fact that the wind resource and operation are under UK control. However the system's dependence on wind power introduces risks around periods of still air (little or no power produced) and the threat of altered resource due to climate change. Perhaps, for this type of power system, improved and increased electricity storage may be an attractive solution to the issue of resilience. Yet, the DREAD scenario actually employs less storage than the LC scenario. As an example, the level of plug-in hybrid vehicles by 2035 in DREAD is only about 60% of the total used in the LC scenario. Yet, this decrease in plug-in hybrid storage in DREAD is unlikely to be a direct result of changing storage requirements. Rather, it is likely that electricity has become so expensive that the model finds other, more cost-effective, options (biofuels ethanol and biodiesel) to decarbonise the transport sector.

This appears to be an example of the limitations of the MARKAL model in that it seems unable to adequately capture the opportunities of various storage technologies to support intermittent power sources. In part, it is due to the lack of temporal detail, the model combines everything into five-year time steps with no annual, seasonal or diurnal structure so is not capable of representing the dynamics of supply and demand realistically; it cannot model a moment when the wind does not blow. It is also perhaps compounded by the fact that the model does not allow for changes to the way that the grid could operate in the future (such as smart grids) and the fact that there is not enough data for advanced storage technologies to be able to model their potential effectively. This suggests the need for further research into the possibilities of accelerated development of storage technologies to contribute to a low carbon and resilient energy system. The work done by UKERC on accelerated technology development has not yet explored accelerated development of storage technologies [5]. A similar issue is likely to arise where the model selects nuclear and wind, as nuclear cannot be ramped up and down to balance supply.

Another interesting technology shift can be seen upon closer inspection of the types of wind power deployed in the DREAD scenario. For the first time in any of the UKERC scenarios, wind micro-generation is introduced to the electricity system. DREAD deploys 5 GW of wind micro-generation. It seems likely that this only reaches 5 GW due to the very low capacity credits for any micro-wind over a total of 5 GW. The model has a total constraint on micro-wind power of 15 GW [57]. Micro-generation with wind is unattractive for national provision as it has high costs and low capacity. Its deployment that there are significant difficulties in achieving decarbonisation in the electricity sector under the constraints imposed by the DREAD scenario.

While NIMBY and ECO show less dramatic technology shifts than DREAD, there are still important changes in the electricity system. In NIMBY, where coal CCS is constrained to only a few GW of installed capacity, nuclear power and wind are able to relatively easily generate the majority of the power needed by 2050 (despite being modestly constrained themselves). However, this does require NIMBY to have a larger system in terms of installed capacity than the LC scenario. The ECO scenario also deploys high levels of wind and nuclear but by 2050 requires higher levels of electricity than the other scenarios due to additional electrification of other sectors. The increased electrification could be due to the increased cost of fossil fuels which make electrification a more economical choice.

Other interesting shifts can be seen in the different ways that limited bioenergy resources are utilised in NIMBY and ECO (the two scenarios in which bioenergy is constrained). In NIMBY, the limited bioenergy resources are shifted to the aviation sector where they are used to make bio-kerosene. While bio-kerosene is never deployed in the LC scenario (or any other core scenario investigated by UKERC Energy 2050), both the NIMBY and DREAD scenarios replace all aviation jet fuel with bio-kerosene by 2050. In the ECO scenario, in which bioenergy is very heavily constrained through the prohibition of imported crops as well as transport biofuels, the allowable bioenergy is still deployed. In ECO, the limited biomass that is still available is utilised in the service sector in the form of wood and later pellets. These shifts and the continued use of bioenergy despite constraints highlight how the flexibility of bioenergy resources can help the system achieve decarbonisation—even in the light of possible constraints on sourcing and usage.

The third key element of the scenario's different strategies to achieve decarbonisation under possible socio-environmental sensitivities is varying levels of demand reduction. As noted in Section 2, UK MARKAL MED allows for flexible demand reduction in response to energy prices, optimising the balance between the cost of supplying energy and the lost welfare incurred by any reduction in energy service demand. As with the various sources of energy generation, levels of demand reduction can be tracked at 5-year intervals throughout the period of the model. The level of demand reduction reported at any given five-year time step relates to the cost of supplying energy services at that point in time. Years where the available energy supply technology mix is more expensive will drive greater demand reduction than in years where the cost of supplying energy is less. Thus, the trajectory of demand reduction is not smooth but responds dynamically to the cost of supplying energy within the steadily increasing carbon constraint. As a consequence, the outputs do not appear as simple smooth lines but can change direction in consecutive time periods.

**Figure 4:**
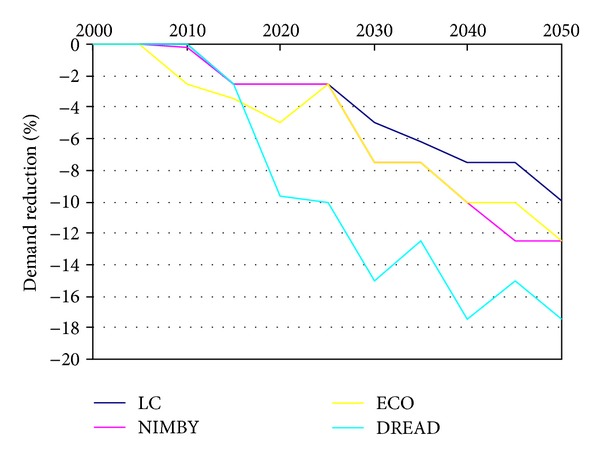
Residential Electricity Demand Reduction.

**Figure 5:**
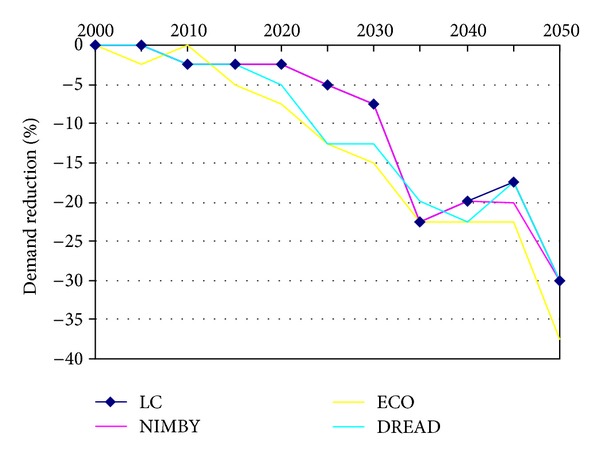
Residential gas demand reduction.

In all scenarios, including the LC scenario, there is a general trend for increasing need to reduce demand over time, reflecting the generally increasing cost of the supply mix, which is in turn driven by the increasing carbon constraint. Yet, for all three socio-environmental scenarios, the additional constraints on the system arising from the limits placed on key technologies cause some level of increased demand reduction across different sectors of the energy system (such as industry residential, and transport). The varying level of demand reduction depends in part on the relative costs of demand reduction and decarbonising power generation. As an example, the demand reduction in residential electricity and gas (Figures 4 and 5) shows an increasing trend over time as well as increased demand reductions in the socio-environmental scenarios.

In general, the NIMBY scenario's demand reduction is only greater than the LC scenario in certain sectors and time periods. Thus, NIMBY could be considered to employ roughly similar (albeit slightly increased) demand reduction strategies to the LC scenario. Yet, in ECO and DREAD, the level of demand reduction utilised is generally greater than that in the LC scenario in all sectors and across most of the time periods. This suggests that ECO and DREAD constraints may increase the stress on the system more than the NIMBY constraints; alternatively, the costs of generating low carbon power are higher than those associated with reducing demand.

The greatest deviation from the standard LC scenario is the use of residential electricity in DREAD (Figure 4). The rate of change is the highest between 2010 and 2020 by which time DREAD's demand reduction has already increased to the level achieved in LC by 2050. This reflects the added cost, especially in the early periods, of making the low carbon transition whilst excluding technologies such as nuclear and CCS. All four scenario variants then change at approximately the same rate. For residential gas, the four scenario variants change more consistently (Figure 5).

### 4.2. Costs of Socio-Environmental Constraints

All three socio-environmental scenarios explored in this paper impose higher financial and social costs than the LC scenario. The increased costs can be seen in three different measures: marginal cost of CO_2_, undiscounted energy system cost, and consumer and producer surplus. Consumer and producer surplus is used as a measure of societal welfare and is thus an indicator of the scenarios' social costs.

**Figure 6:**
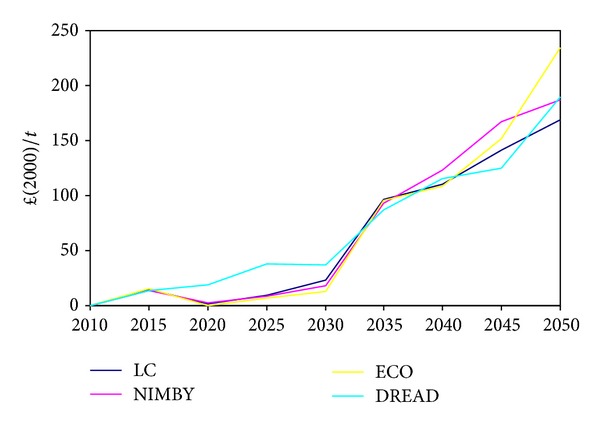
Marginal cost of CO_2_.

The marginal cost of CO_2_ increases over time in all of the scenarios, including the baseline LC scenario, due to the increasing difficultly and cost of achieving the decarbonisation targets (80% by 2050). However, the marginal cost in some of the socio-environmental scenarios increases at a faster rate or at different times than the LC scenario. In the near term (2015–2030) the DREAD scenario has the highest marginal cost of CO_2_. However, by 2050 the marginal cost of CO_2_ in the ECO scenario is the highest and is rapidly increasing as seen in Figure 6.

The undiscounted energy system cost can be used as another measure of the financial costs of the socio-environmental scenarios. It is roughly similar in the LC, NIMBY and DREAD scenarios—although NIMBY and DREAD each deviate upwards from the LC scenario at certain times. In the ECO scenario, however, the undiscounted energy system cost is consistently higher than any of the other scenarios from 2015 onwards. This increased cost in ECO grows from approximately *£*2.5 billion more expensive than the next most expensive scenario in 2015 to *£*9 billion more in 2050. This suggests that the ECO constraints force the model to do much more of the expensive decarbonisation measures in order to be able to achieve decarbonisation targets. Given that MARKAL optimises the energy system for the lowest energy system costs, ECO would appear to cause the highest level of marginal costs associated with CO_2_ on the system.

**Figure 7:**
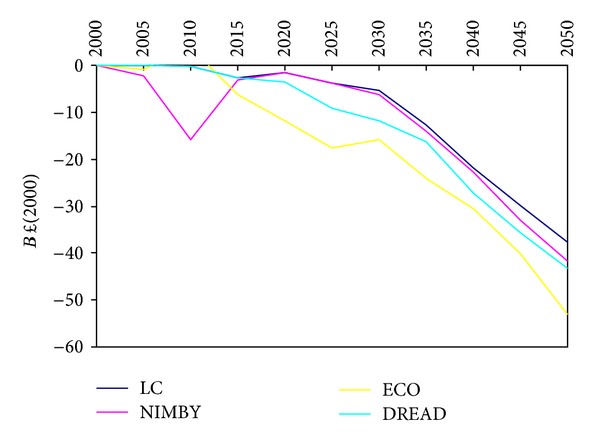
Societal welfare expressed as consumer and producer surplus.

If the sum of consumer and producer surplus is used as a partial measure of social welfare, it becomes evident that all of the socio-environmental scenarios impose a higher social cost than the LC scenario. Yet, it is the ECO scenario that once again imposes the greatest costs; the ECO scenario shows a significantly greater decline in welfare from 2015 onwards than the LC, NIMBY, or DREAD scenarios (Figure 7). However, this measure of social welfare is perhaps not the best way to envision social welfare in these types of scenarios. The constraints in these scenarios are meant to represent social sensitivities and preferences, so there should be some social benefits gained by responding to these preferences. Consumer and producer surplus, a traditional measure of welfare (which theorises decreasing social welfare with increasing demand reduction, etc.), may not be as applicable in a possible future where people choose to reduce demand and remake the energy system in response to concerns and sensitivities.

These three measures illustrate that public responses to low carbon technologies have the potential to substantially impact not only the make-up of the energy system but also the financial and social costs of decarbonisation. The way that public attitudes and responses constrain the deployment of certain technologies or resources could make decarbonisation more challenging and costly but it could also have the potential to make decarbonisation more equitable and just. Yet, these socio-environmental sensitivities are not always included in discussions of decarbonisation; unfortunately, when they are, the focus is often on overcoming public attitudes rather than genuinely engaging with and considering public sensitivities. If these public sensitivities and responses continue to be neglected, there may be an increased chance of decarbonisation targets not being met, serious backlash from the public, and/or increased costs of decarbonisation.

## 5. Discussion

### 5.1. Societal Impacts and Interactions

The energy systems which are deployed in the three socio-environmental scenarios are all working systems given the conditions imposed by the MARKAL model and they all reach 80% decarbonisation by 2050. However, in the real world, these energy systems could be difficult to deploy and/or manage and, in some cases, may seem implausible. The level of challenge in these scenarios reflects the severity of the potential socio-environmental constraints and the stresses that those constraints could impose on the system. These stresses could make it difficult to achieve decarbonisation targets while continuing to meet society's demand for energy services. Yet, ignoring these potential socio-environmental constraints does not stop the constraints from arising; it just means being less prepared to deal with them. Thus, if there continues to be a gap in the understanding and methods of working with public sensitivities to low carbon energy technologies and resources, it will just make the issue harder and more expensive to deal with in the future [58].

The scenarios suggest that important changes will need to be made to both the technologies deployed in the energy system as well as to the way the society interacts with issues of energy and climate change. One of the more extreme scenarios, DREAD, is highly dependent on wind power and has little diversity within the electricity sector. Accordingly, DREAD is a costly scenario and would seem to be implausible in reality. However, if society was to take such a risk averse position creating a less resilient energy system, other values and components would have to change. For instance, in DREAD's wind dominated system these necessary changes might include advances in storage, grid technologies, interconnection, thermal backup and demand side response to manage the high level of wind power, public willingness to reduce demand, and/or public acceptance that the full 80% decarbonisation may not be met. This message is likely to be an unpopular one; it highlights the seriousness of the decarbonisation challenge.

Two potential ways to manage an energy system under these types of socio-environmental constraints include technological solutions and a re-evaluation of priorities and choices (this is a more societal solution that means involving the public in choices). These options are by no means mutually exclusive. In terms of technological solutions, there is potential for advances in certain technologies (such as storage) and improvements in grid infrastructure and operation (such as smart grids) to make the scenarios more feasible. Technological fixes are, however, unlikely to be the only answer. This work also highlights the importance of genuinely considering and engaging with socio-environmental sensitivities; societies may need to consider their priorities and options (on a personal and societal level) and make some difficult choices.

It is important to note that genuinely addressing socio-environmental sensitivities and public opinions does not mean ignoring or overcoming them, rather it suggests that they need to be understood and incorporated into decisions. Unfortunately, as Devine-Wright [59, page 10] states, “genuine understanding of the dynamics of public acceptance remains elusive.” Working with Batel [60] they call for the concept of “acceptance” to be debated and redefined in terms of public attitudes towards low carbon technology and its infrastructure. Aitken [61] suggests the need to better understand the social context of renewables as well as the public relationship with planning processes. Likewise, Ellis et al. [62] point out that this understanding should not just be focused on objectors, as this completely misses out on any understanding of the way that support is constructed.

Decisions about the energy system currently seem to be made whilst leaving the public a bit behind, viewing the public as a barrier to be overcome or an enabler of certain types of action rather than inherently the core group of decision makers. This research suggests that the public actually have a more important and involved role to play because ultimately all members of society will determine the future of the energy system through the decisions they make and the way that they interact with technologies, institutions, and policies. Thus, this work calls for an increased understanding of public sensitivities around low carbon technologies and changes to the process of energy system change in order to get the public engaged and incorporated early in the process of decarbonisation. This could take the shape of new methods of public engagement, alternative models of community ownership and/or benefits, different methods of studying public sensitivities, changes in planning procedure, and/or changes to the way that the challenges of decarbonisation and the necessary choices and trade-offs are shared with and discussed with the public.

If this does not happen early in the process of managing decarbonisation and there remains little understanding of socio-environmental sensitivities and poor public engagement, there may be subsequent choices which do not reflect prevailing social sentiments. This is likely to make decarbonisation even more challenging as there would be less time to prepare more acceptable, alternative solutions.

There is a need for further research on how best to engage with the complex area of real public socio-environmental sensitivities; scenarios such as this one are too simplified to accurately reflect the interactions of broad socio-environmental sensitivities but nevertheless attempt to begin exploring the issue using a highly utilised modelling tool.

## 6. Conclusions

These socio-environmental scenarios have illustrated how potential socio-environmental sensitivities and public acceptance of technologies could impact the possible pathways to decarbonisation and the energy system as a whole. Despite the fact that the scenarios explored in this work cannot fully represent the complexities of the real world and are not designed to be forecasts, they do offer useful insight into the potential types of impact on the energy system. The scenarios indicate that socio-environmental sensitivities could play a significant role in shaping how decarbonisation could be achieved, and at what cost.

Accelerating the development of a suite of low carbon energy technologies could be an important way to improve the chances of achieving the decarbonisation targets given a potentially socio-environmentally constrained energy system. As it is difficult to predict how society will respond to low carbon energy technologies and the changing energy system, having a number of alternatives available could help ensure that there are some acceptable options to achieve decarbonisation.

These scenarios also suggest the need for further research which could better represent the complexities of socio-environmental sensitivities and public acceptance. In reality, attitudes and acceptance are multi-faceted issues, vary immensely amongst the members of the public, and are not static over time; this makes it impossible to predict or reflect public acceptance issues with any degree of accuracy. Yet, by modelling a few simplified scenarios of possible socio-environmental sensitivities, this work is able to begin to explore the different types (and severity) of impacts that could be seen in the energy system. With hope, the results from the scenarios can be used to better inform the process of decarbonisation by raising important issues about how members of the public are engaged with low carbon technologies and the process of wider energy system change for decarbonisation; these issues must be considered in order to improve the chance of achieving decarbonisation targets.
